# Serial crystallography at synchrotrons and X-ray lasers

**DOI:** 10.1107/S2052252517001877

**Published:** 2017-02-23

**Authors:** Jörg Standfuss, John Spence

**Affiliations:** a Laboratory of Biomolecular Research, Division of Biology and Chemistry, Paul Scherrer Institute, Villigen PSI, Switzerland; b Department of Physics, Arizona State University, Tempe, Arizona, USA

**Keywords:** serial crystallography, synchrotrons, X-ray lasers, editorial

## Abstract

Serial crystallography was developed for the use at free-electron lasers but the approach has recently also been adapted to synchrotron sources. Here we discuss how the synergy between the two X-ray sources will facilitate a wide application of the technique in microcrystallography, room-temperature structure determination and time-resolved studies.

Recently we have seen rapid progress in the serial crystallography (SC) method at X-ray free-electron lasers (XFELs). Injection of thousands of protein microcrystals into the ∼10^12^ photons of few-femtosecond XFEL pulses has allowed the structure determination of crystals grown *in vivo*, or of submicron size, and from challenging targets such as membrane proteins. For time-resolved studies, the small crystal size allows for rapid diffusive saturation in mix-and-inject analysis of biochemical reactions, and full optical saturation of the sample by a pump laser in studies of light-driven proteins. The ability to outrun most radiation damage avoids the need for sample cooling and its artifacts, allowing studies of molecular machines at work in their correct room-temperature thermal bath or a controlled chemical environment.

Despite these achievements, the XFEL community remains relatively small, due to the limited availability of XFEL beamtime which, at present, allows only two simultaneous data collections worldwide (in Japan and USA). In addition, serial femtosecond crystallography (SFX) at XFELs is experimentally demanding and complex, currently requiring sizable interdisciplinary teams of collaborating scientists and engineers. The transfer of the SC approach to next-generation synchrotrons upgraded for higher flux density and with beamlines using sophisticated focusing optics, submicron beam diameters and fast low-noise photon-counting detectors offers a way out of this dilemma. In applications such as room-temperature data collection or phasing from radiation-sensitive microcrystals, serial millisecond crystallography (SMX) at synchrotrons has developed into a viable alternative. In the near future, it may even be possible to extend the method to time-resolved studies. Already it frees beamtime at XFELs to exploit their unique capabilities, such as outrunning damage in structure analysis from nanocrystals or ultrafast time-resolved crystallography.

The experimental origins of fast SC can be traced to an early proposal to fire bioparticles in a continuous single-file stream across a beam for diffraction analysis using a Rayleigh jet (Spence & Doak, 2004 ▸) and the first tests of protein microcrystals in such a system at the Advanced Light Source (Shapiro *et al.*, 2008 ▸), in preparation for use at the world’s first XFEL, the LCLS at SLAC, in the first SFX experiments (Chapman *et al.*, 2011 ▸). In SFX, each successive nano- to micrometre-sized crystal is destroyed by the beam pulse, after providing a high-resolution set of Bragg peaks which outrun radiation damage. The problem of merging partial reflections (and estimating the degree of partiality from X-ray snapshots) was analyzed by Bolotovsky *et al.* (1998 ▸). Rossman has described this as ‘the American Method – shoot first and ask questions later’, since in SC, in the absence of a goniometer, the orientation of the crystal (perhaps of unknown structure) and the diffraction conditions must be found by software. **IUCrJ** now (http://journals.iucr.org/m/services/readerservices.html) shows many papers on SC, with some of the most cited examples devoted to the interplay between synchrotrons and XFELs.

The term ‘serial’ in synchrotron studies has recently become popular in a wider sense, and may also refer to taking a series of diffraction patterns along a few larger crystals, or the scanning of fixed target devices. Such serial approaches share the idea of distributing the radiation dose, to get the maximum signal from all of the available crystal volume. Short millisecond exposures may outrun slower secondary radiation damage from diffusing radicals or relaxation of individual damaged molecules (Warkentin *et al.*, 2013 ▸). Injection-based methods, well suited to the next generation of diffraction-limited sources, take full advantage of these effects by collecting snapshots from thousands of continuously replenished tiny crystals to reconstruct structures at room temperature.

An important application where injector-based SC has proven advantageous is fast time-resolved pump–probe crystallography. Myoglobin (Barends *et al.*, 2015 ▸) and the photoactive yellow protein (Pande *et al.*, 2016 ▸) have provided success stories of how the technique can be applied to bio­logical problems, the second case giving us a remarkable molecular movie (at 200 fs time resolution) of the primary photochemical *cis*–*trans* reaction. Yet both proteins are available in large amounts and can be grown into big crystals suitable for time-resolved Laue diffraction experiments at a synchrotron. A recent study of the light-driven proton pump bacteriorhodopsin, for which only a few micrometre thick crystals were available, resulted in a series of 13 snapshots at logarithmically spaced time points after activation (Nango *et al.*, 2016 ▸). By eliminating concerns about radiation damage and providing a consistent molecular movie from nanoseconds to milliseconds, this work resolves a lengthy debate on inconsistencies in synchrotron freeze-trapping experiments. The movie furthers shows in remarkable detail how energy from the initially twisted retinal chromophore is transferred into rearrangements of the protein, a reaction nature has adapted to a wide range of biological functions including our visual sense.

The work was only feasible using sample-efficient high-viscosity injectors (Weierstall *et al.*, 2014 ▸) to reduce the amount of protein needed. Such ‘toothpaste jet’ sample-delivery devices and fast low-noise X-ray detectors offer major advantages for SC using synchrotron sources. It has now become possible to record millisecond exposures which are briefer than the rotational diffusion time of the microcrystals in a viscous delivery medium (such as LCP, PEG or agarose). Diffraction conditions change by a negligible amount during this time, so that efficient SC with a continuous flow of sample becomes possible (Nogly *et al.*, 2015 ▸; Botha *et al.*, 2015 ▸) and may be developed into a routine method for room-temperature structure determination. Initial bacteriorhodopsin crystals were tested by SMX at a synchrotron and by SFX at an XFEL, a good example of the synergy between the two X-ray sources (Nogly *et al.*, 2016 ▸). Currently, an injector-based time-resolved experiment at synchrotron beamlines would be limited to a time resolution of several milliseconds, as this is the time needed to collect diffraction patterns with sufficient intensity. Future synchrotron upgrades to next-generation diffraction-limited sources will allow measurements in the micro- and perhaps nanosecond range for study of the vast majority of the slower processes which occur in biology. Artificial photoswitches and mix-and-inject methods seem bound to spread the application of SC further at both synchrotrons and XFELs, allowing chemical triggers to be used in the study of processes such as enzyme/substrate reactions and drug binding. It seems clear that the interplay between XFELs and synchrotrons will be an important factor in keeping the century-old improvements in the crystallographic method advancing. Time will tell which approach is best suited to particular biological systems and problems, while both radiation sources will benefit from these synergistic developments.
